# Delivering Singlet Oxygen in Dark Condition With an Anthracene-Functionalized Semiconducting Compound for Enhanced Phototheranostics

**DOI:** 10.3389/fbioe.2022.781766

**Published:** 2022-03-09

**Authors:** Jian Shen, Liuhong Pan, Xujing Zhang, Zhenyuan Zou, Bo Wei, Yongchang Chen, Xiaoyan Tang, Dengfeng Zou

**Affiliations:** ^1^ Department of Urology, Changshu No. 2 People’s Hospital, Changshu, China; ^2^ School of Pharmacy, Guilin Medical University, Guilin, China; ^3^ Department of Materials Engineering, Changshu Institute of Technology, Changshu, China

**Keywords:** heavy-atom–free, DPPA, human kidney cancer, fractionated PDT, synergistic therapy

## Abstract

Photodynamic therapy (PDT) utilizes the photogeneration of reactive oxygen species (ROS) with high cytotoxicity to kill cancer cells, holding great promise for cancer treatment. Fractionated delivery of singlet oxygen (^1^O_2_) is a wise approach to relieving hypoxia, thus enhancing the therapeutic efficacy. In this article, an anthracene-functionalized semiconducting compound (DPPA) has been designed and synthesized. With irradiation, the compound is able to undergo efficient intersystem crossing (ISC) and non-radioactive decay for photodynamic/photothermal synergistic therapy. In addition, the anthracene module is able to capture and release ^1^O_2_ reversibly with or without irradiation. DPPA nanoparticles (NPs) obtained by nanoprecipitation with DSPE-PEG exhibit considerable high phototoxicity on human kidney cancer cells (A498), and the half maximum inhibitory concentration (IC_50_) is 15.8 μg/ml. Furthermore, an *in vivo* study demonstrates that complete tumor suppression was observed when the mice were administered DPPA NPs with the help of laser, compared with the control and dark groups. The H&E analysis of the normal tissues (the heart, liver, spleen, lungs, and kidney) indicates that such NPs cause no side effects, indicating the biosafety of DPPA NPs. The results provide a strategy to design a heavy-atom–free photosensitizer for photothermal and fractionated PDT against kidney tumors.

## Introduction

With the increasing cases of cancer worldwide, the development of new therapeutic methods for cancer treatment is of tremendous significance. (Siegel et al., 2021). Phototherapy utilizes the photogeneration of cytotoxic reactive oxygen species (ROS) (Xu et al., 2017; Zhen et al., 2017; Huang et al., 2018; Li et al., 2019a; Liu et al., 2019a; Li et al., 2019b; Meng et al., 2019; Li et al., 2020; Xiao et al., 2020; Yang et al., 2020; Yao et al., 2020; Zheng et al., 2020; Zou et al., 2021a; Zou et al., 2021b; Wang et al., 2021) or heat (Chen et al., 2019; Li et al., 2019c; Ma et al., 2019; Zhang et al., 2019; Zhao et al., 2020) to induce cell apoptosis and further leads to tumor suppression, holding great promise for cancer treatment. (Zhou et al., 2016; Ng et al., 2018; Liu et al., 2019b). However, in the photoinduced ROS generation, especially the oxygen-dependent type II process, continuous irradiation will inevitably cause hypoxia, which will, in turn, reduce the oxygen supply and diminish the therapeutic efficacy. (Fan et al., 2016). Therefore, hypoxia is acknowledged as the obstacle of photodynamic therapy (PDT).

Continuous irradiation of the tumor leads to the burst release of ROS and induces tumor hypoxia, which is disadvantageous for cancer treatment. Fractionated delivery of singlet oxygen in the dark environment may be a wise strategy to enhance the therapeutic efficacy. (Turan et al., 2016; Zhu et al., 2019; Zou et al., 2020). It is considered as a mild PDT process, resulting in the diminished blood vessel damage and providing enough time for the oxygen supply in the blood circulation. Anthracene derivatives are capable of capturing singlet oxygen to form an endoperoxide intermediate by a cycloaddition reaction with laser irradiation. Furthermore, in the dark cycle, the endoperoxide will reversibly release ^1^O_2_ to regenerate the anthracene modules. (Wang and Zhao, 2017; Filatov et al., 2018; Zhu et al., 2019). In addition, anthracene derivatives are usually considered as heavy-atom–free compounds for efficient intersystem crossing (ISC), and the dark toxicity may be quenched. (Filatov et al., 2017; Callaghan et al., 2019). Considerable attention has been attached to semiconducting compounds due to their unique photophysical and photochemical properties. (Chen et al., 2016; Tang et al., 2018; Li et al., 2019d; Shen et al., 2019; Deng et al., 2020; Zhang et al., 2021). For example, *Chen* et al. designed a heavy-atom-free compound for efficient singlet oxygen generation and continuous PDT. (Zou et al., 2020). Another example is that *Pu* et al. designed a semiconducting polymer for PDT-induced immunotherapy. (Li et al., 2019e).

In this work, we have designed and prepared a heavy-atom-free semiconducting compound 3,6-bis[5-(anthracen-9-yl)furan-2-yl]-2,5-bis(2-octyldodecyl)pyrrolo [3,4-c]pyrrole-1,4(2H,5H)-dione (denoted as DPPA) by a C-H activation reaction (Scheme). Compared with the standard substance methylene blue (MB), the singlet oxygen quantum yield (^1^O_2_ QY) of the as-obtained DPPA is 21.3% in dichloromethane (DCM). DPPA nanoparticles (NPs) obtained by nanoprecipitation exhibit spherical morphology with an average diameter of 52 nm. Such NPs are able to capture singlet oxygen with irradiation and release it in the dark condition. The photothermal conversion efficiency of DPPA NPs is 35.6%. The PDT and PTT synergistic effect may promise the excellent therapeutic efficacy of DPPA NPs. (Wang et al., 2019; Wu et al., 2019; Chang et al., 2020; Xu et al., 2020). *In vitro* MTT assay indicates the half maximum inhibitory concentration (IC_50_) of DPPA NPs is as low as 15.8 μg/ml in human kidney cells (A498) with laser irradiation. Further fluorescence imaging *in vivo* suggests that DPPA NPs are able to passively target the tumor by the EPR (enhanced permeability and retention) effect. With the help of a laser, DPPA NPs are capable of inhibiting the tumor growth while exerting little side effects on normal tissues, including the heart, spleen, liver, kidney, and lungs. The results suggest that DPPA NPs have great potential for photothermal and fractionated photodynamic therapy.

## Experimental Section

### Materials and Apparatus

^1^H NMR and ^13^C NMR spectra were performed on a Bruker DRX NMR spectrometer in CDCl_3_ (*δ* = 7.26 ppm) at 298 K as the internal standard. UV-vis and fluorescence spectra were measured on a Shimadzu spectrophotometer, from Japan, (UV-3600) and a HITACHI spectrometer (F-4600, Japan), respectively. TEM of the nanoparticles were measured on equipment (JEOL JEM-2100). Dynamic light scattering (DLS) of DPPA NPs was tested on a particle size analyzer (90 Plus, Brookhaven Instruments, United States). Fluorescence imaging of DPPA NPs in nude mice was recorded on an IVIS spectrum.

### Synthesis and Characterization of DPPA

A mixture of DPP (200.0 mg, 0.24 mmol), 9-bromoanthracence (160.0 mg, 0.60 mmol), pivalic acid (20 mg, 0.20 mmol), Pt (OAc)_2_ (11.0 mg, 0.02 mmol), and K_2_CO_3_ (83.0 mg, 0.60 mmol) was dissolved in 5 ml DMA (N,N-dimethyl acetamide). Then, N_2_ was bubbled to drive off possible oxygen and water in the system. The mixture was heated to 110°C under the protection of N_2_ gas for 12 h. After cooling to room temperature, the mixture was poured into saturated sodium chloride solution (150 ml) and extracted with dichloromethane (100 ml) three times. The organic layer was washed with brine, followed by drying with anhydrous sodium sulfate. The solvent was removed by rotary evaporation and purified by silica gel column chromatography with dichloromethane and hexane (1: 2, *v/v*) as the developing solvent. Dark blue solids were obtained (Yield: 95 mg, 30%). ^1^HNMR: *δ* H 8.60-8.56 (2H, m), 8.55-8.51 (4H, d), 8.22-8.17 (4H, d), 8.12-8.01 (4H, m), 7.76-7.66 (4H, m), 4.36-4.23 (4H, d), 2.12-2.02 (2H, s), 1.33-1.21 (50H, m), and 0.96-0.76 (26H, m). ^13^CNMR: 160.12, 143.83, 128.53, 127.70, 125.67, 124.55, 115.38, 57.13, 45.73, 37.35,30.84, 25.07, 21.63, 17.31, and 13.09. MS: m/z: 1,180.80, found: 1,181.85.

### Cell Culture and MTT Assay

At 37°C, human kidney cancer (A498) cells were cultured with a medium consisting of 12% fetal bovine serum (FBS) in DMEM (Gibico) under the atmosphere of 5% CO_2_. DPPA NPs with different concentrations were co-cultivated with A498 cells in the 96-well plate. For the illumination group, each well was irradiated with a 660 nm laser for 8 min. In contrast, the wells in the control and no illumination groups have not been irradiated. Relative cell viability was determined by recording the absorbance of MTT [3-(4,5-dimethylthiazol- 2-yl)-2,5- diphenyltetrazolium bromide]. MTT in PBS (5 mg/ml) was added to the well (20 μl) and then incubated for 4 h. After that, the mother liquid was discarded, and DMSO (200 μl) was added. The absorbance of each well was recorded on a Bio-Tek microplate reader. Cell viability was then calculated according to the equation:

Cell viability (%) = mean absorbance of the group incubated with DPPA NPs/mean absorbance of the group.

All the cell experiments were repeated three times.

### Cellular Uptake and Fluorescence Imaging of Cellular ROS

A498 cells were cultured with DPPA NPs (3 ml) in a confocal dish for 4 h. Then, the medium was discarded, and the cells were washed with PBS (1 ml, 3 times), followed by the co-culture with 1 ml polyoxymethylene for 25 min. Then, polyoxymethylene was discarded, and the cells were also washed with PBS three times (1 ml). The cells were further co-cultivated with DCF-DA (2,7-dichlorodihydrofluorescein diacetate, 10 µmol) for 5 min, followed by washing with PBS (1 ml) three times. A 660 nm laser was then applied to the sample for 3 min (0.5 W/cm^2^). The cells were excited at 633 nm, and fluorescence was observed from 650 to 750 nm to investigate the cellular uptake. They were excited with a 488 nm laser, and fluorescence was observed from 490 to 560 nm to show the ROS generation.

### Fluorescence Imaging- and Photothermal Imaging-Guided Phototherapy

The procedure follows the rules of the National Institutes of Health (NIH). The animal study was approved by Guilin Medical University (SCXK 2007-001). A total of 15 nude mice were purchased and then inoculated with A498 cells. Three mice have been chosen to perform *in vivo* fluorescence imaging. The fluorescence image was captured first, and then, three mice were intravenously injected with DPPA NPs, and the fluorescence imaging pictures were also captured at different time points. A total of 12 nude mice were divided into three groups at random when the tumor volume reached about 80 mm^3^. For the dark and illumination groups, the mice were intravenously injected DPPA NPs (200 μg/ml, 100 μL). After 12 h, the tumors of the PBS + laser and DPPA + laser groups were irradiated by a 660 nm laser (1 W/cm^2^) for 8 min, while the mice in the DPPA-only group were not irradiated. These nude mice were then sacrificed for histology analysis.

### Statistical Analysis

All numeric data are expressed as mean ± s.d., unless otherwise indicated. The significance between two groups was analyzed by the two-tailed Student’s t-test. Statistical analysis was performed by GraphPad Prism 6.0. *p* values of less than 0.05 were considered significant (*p < .05, **p < .01, ***p < .001).

## Results and Discussion

### Synthesis and Generation Characterization of DPPA and NPs

**FIGURE 1:**
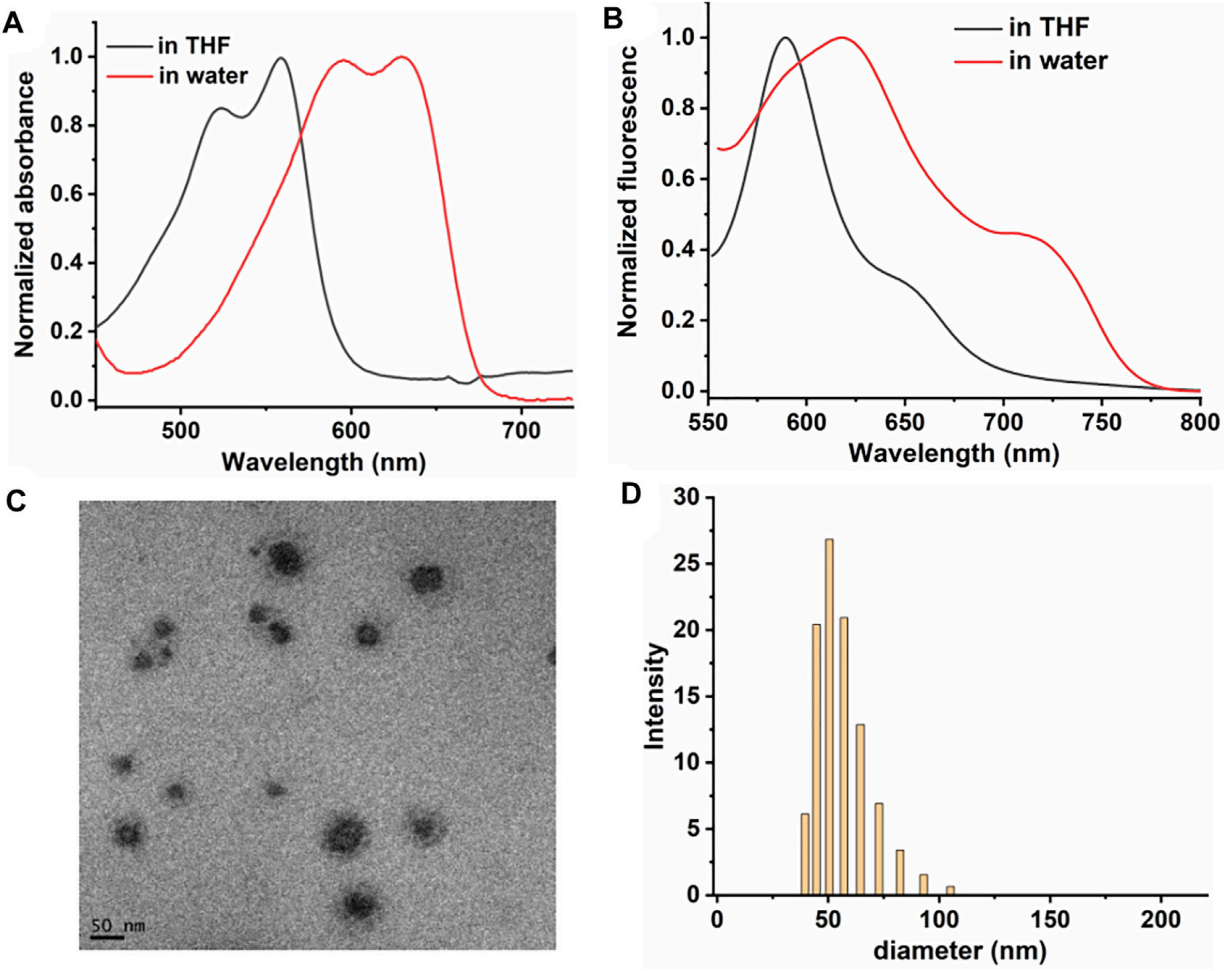
Normalized **(A)** absorbance spectra of DPPA. **(B)** fluorescence spectra of DPPA in THF and NPs in water. **(C)** TEM of DPPA NPs. **(D)** DLS of DPPA NPs in water.

DPPA was prepared and characterized by ^1^HNMR, ^13^CNMR, and mass spectroscopy (Supplementary Figure S1–S3). DPPA NPs were characterized by the UV-vis and fluorescence emission spectra. DPPA shows narrow absorption peaks at 522 and 558 nm in DCM, while the emission peaks were shifted to 593 and 629 nm, respectively, indicating their responsiveness to near-infrared (NIR) light. (Figures 1A,B). A large Stokes shift was observed for the absorbance of the maximum absorbance of DPPA NPs in water (616 and 719 nm), which is attributed to both the solvent effect and the aggregation of DPPA NPs in aqueous solution. This phenomenon could also be found of other photosensitizers. (Zou et al., 2020). The morphology characterized by a transmission electron microscope (TEM) suggests DPPA is self-assembled to form uniform NPs (Figure 1C), consisting with the dynamic light-scattering (DLS) result (mean diameter ∼52 nm) (Figure 1D).

### Singlet Oxygen Generation, Reversible Capture and Release, and Photothermal Conversion Efficiency

**FIGURE 2:**
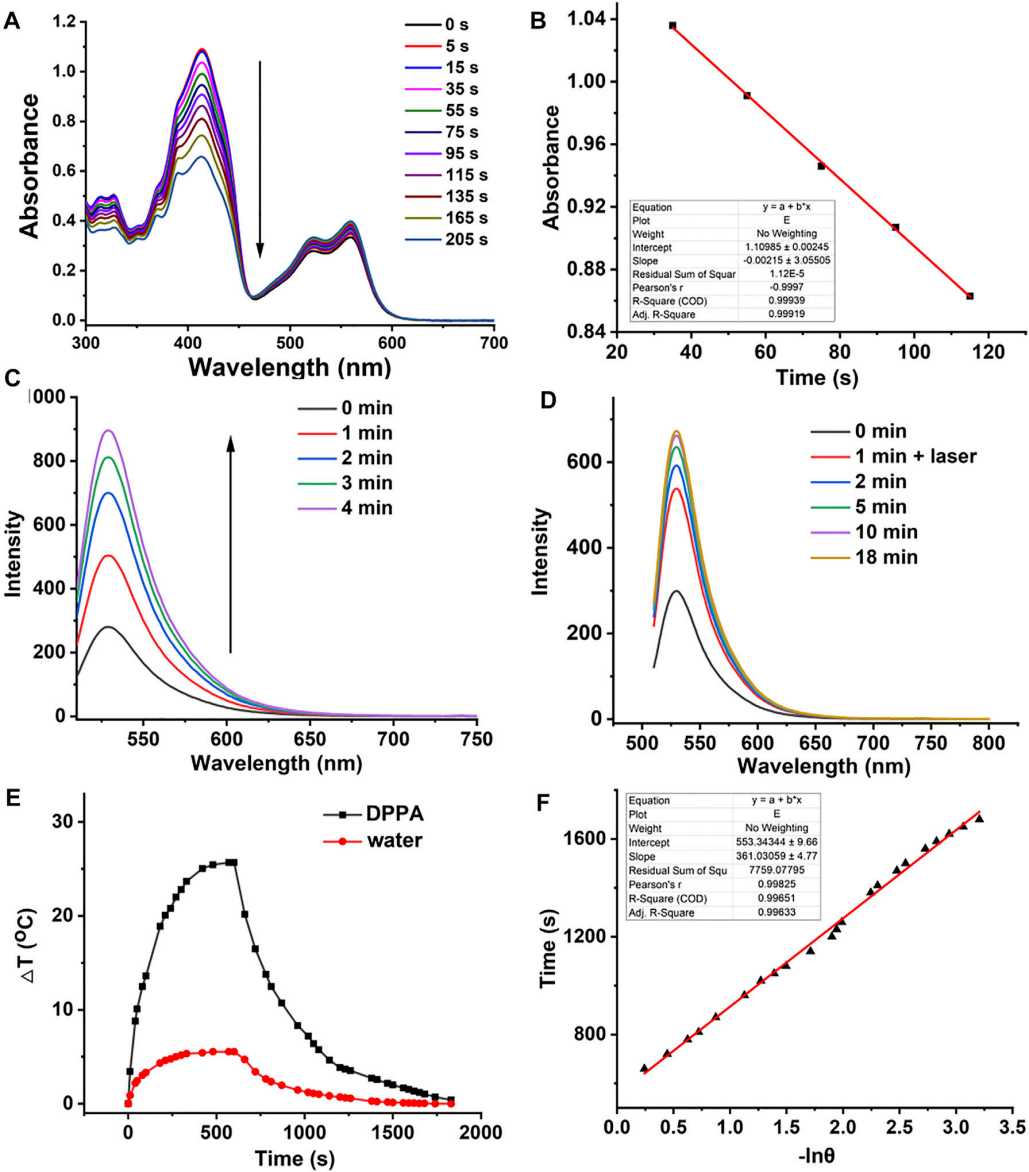
**(A)** ^1^O_2_ generation of DPPA in DCM using DPBF as a probe. **(B)** Linear fitting of time versus absorbance. **(C)** Fluorescence intensity SOSG with laser irradiation (660 nm, 50 W/cm^2^). **(D)** Fluorescence intensity of SOSG with irradiation for 1 min and then without irradiation (660 nm, 50 W/cm^2^). **(E)** Temperature elevation and decrease curve of DPPA NPs (660 nm, 500 W/cm^2^, 10 min). **(F)** Linear fitting of −lnθ versus time.

For an ideal photosensitizer, high singlet oxygen quantum yield (^1^O_2_ QY) promises excellent phototherapeutic efficacy. Therefore, the ^1^O_2_ QY of DPPA was calculated by recording the absorbance of 1,3-diphenylisobenzofuran (DPBF) with laser irradiation. With methylene blue (MB ∼*Φ* = 57%) as the standard substance in DCM (Supplementary Figure S4), the absorbance of DPBF kept decreasing with irradiation while that of DPPA remained unchanged, and the ^1^O_2_ QY is calculated as 21.3% (Figures 2A,B). It is worth noting that DPPA is heavy-atom-free, and this may reduce the potential dark toxicity itself. Then, nanoprecipitation was used to prepare DPPA NPs with good dispersity in water. The singlet oxygen generation ability of DPPA NPs was measured using singlet oxygen sensor green (SOSG) as an indicator. It can be found that DPPA NPs inherit the high ^1^O_2_ generation ability as the fluorescence enhancement of SOSG was enhanced by 3.2 times with irradiation (Figure 2C). However, the singlet oxygen generation ability of DPPA NPs is lower than that of Rose Bengal (Supplementary Figure S6). The ^1^O_2_ capture and release was, then, also characterized by recording the fluorescence intensity of SOSG with or without irradiation. After irradiation for 1 min, the intensity was enhanced two times. It continued to increase even without laser irradiation, indicating the fractionated delivery of ^1^O_2_ in the dark environment (Figure 2D).

High photothermal conversion efficiency promises the photosensitizer with a high photothermal therapeutic efficacy. The heating curve of DPPA NPs in distilled water with irradiation or the cooling curve without irradiation was recorded. (Figure 2E). The temperature elevation of 25.2 C with laser irradiation in the presence of DPPA is much higher than that of water under the same condition (5.1 C) with a high photothermal conversion efficiency of 35.6% (Figure 2F). Such NPs show excellent photostability because no obvious decay was observed, regardless of irradiation (Supplementary Figure S5).

### Cellular Uptake, ^1^O_2_ Generation, and MTT Assay *In Vitro*

**FIGURE 3:**
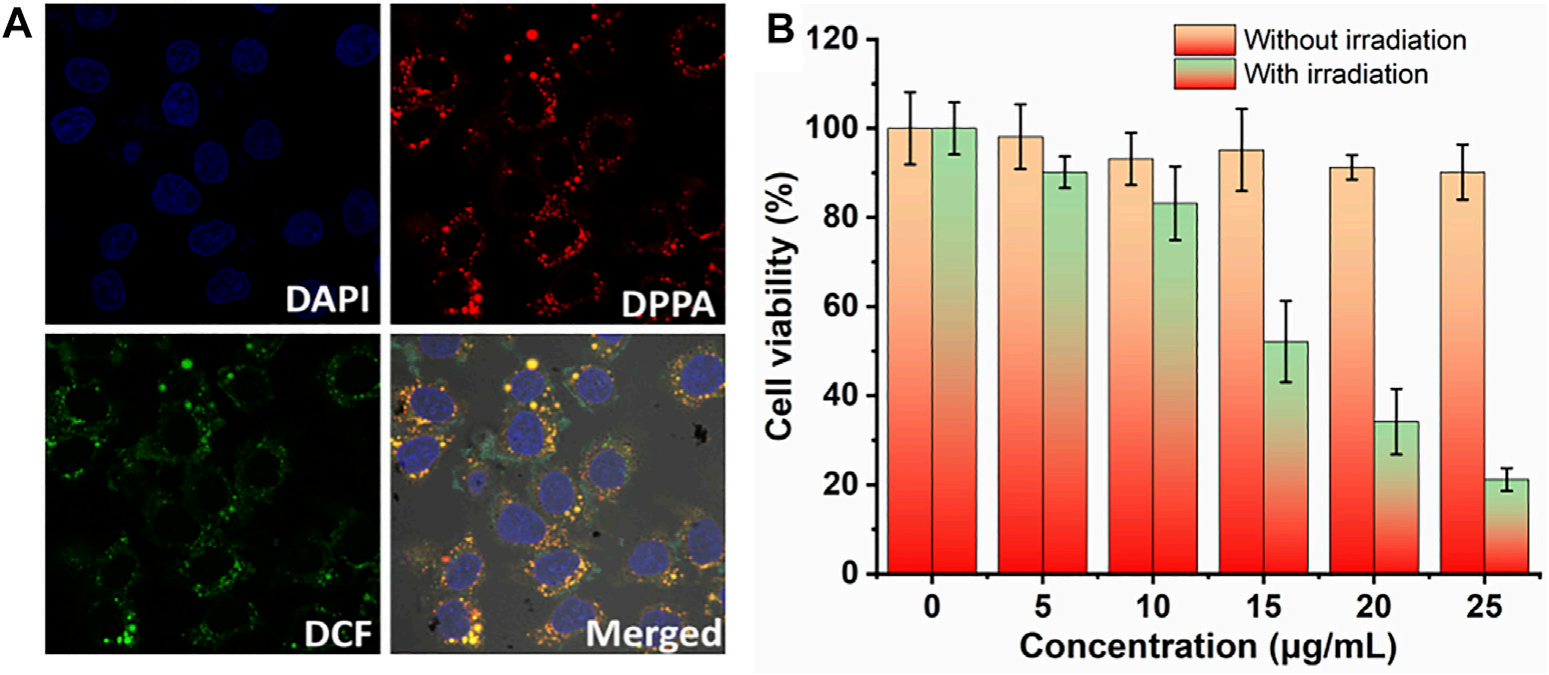
**(A)** *In vitro* cellular uptake of DPPA NPs in A498 cells and ROS generation in the presence of DCF-DA as a probe (660 nm, 100 W/cm^2^). **(B)** Cell viability of A498 cells in the presence of DPPA NPs at different concentrations (0, 5, 10, 15, 20, and 25 μg/ml) (660 nm, 100 W/cm^2^, 8 min).

Based on the singlet oxygen detection and photothermal conversion efficiency investigation, we then evaluated the therapeutic efficacy of DPPA NPs *in vitro*. The cellular uptake and singlet oxygen generation ability of DPPA NPs were investigated by confocal laser scanning microscopy (CLSM). DPPA NPs are able to be uptaken by human kidney cells (A498) after incubation for 6 h (Figure 3A). With laser irradiation, singlet oxygen generation could be observed due to the strong green fluorescence. (Figure 3A). After incubation for 24 h, two groups were divided to investigate the dark or photo toxicity by MTT assay. For the dark group, the cell viability remained very high, regardless of the concentration, indicating the low dark toxicity of DPPA NPs (Figure 3B). In comparison, the cells’ viability with irradiation show concentration-dependent death, and the half maximum inhibitory concentration of DPPA NPs is 15.8 μg/ml (Figure 3B). The results demonstrate that DPPA NPs have a potential for PDT/PTT synergistic therapy.

### *In Vivo* Fluorescence Imaging-Guided Photothermal and Fractionated Photodynamic Therapy

**FIGURE 4:**
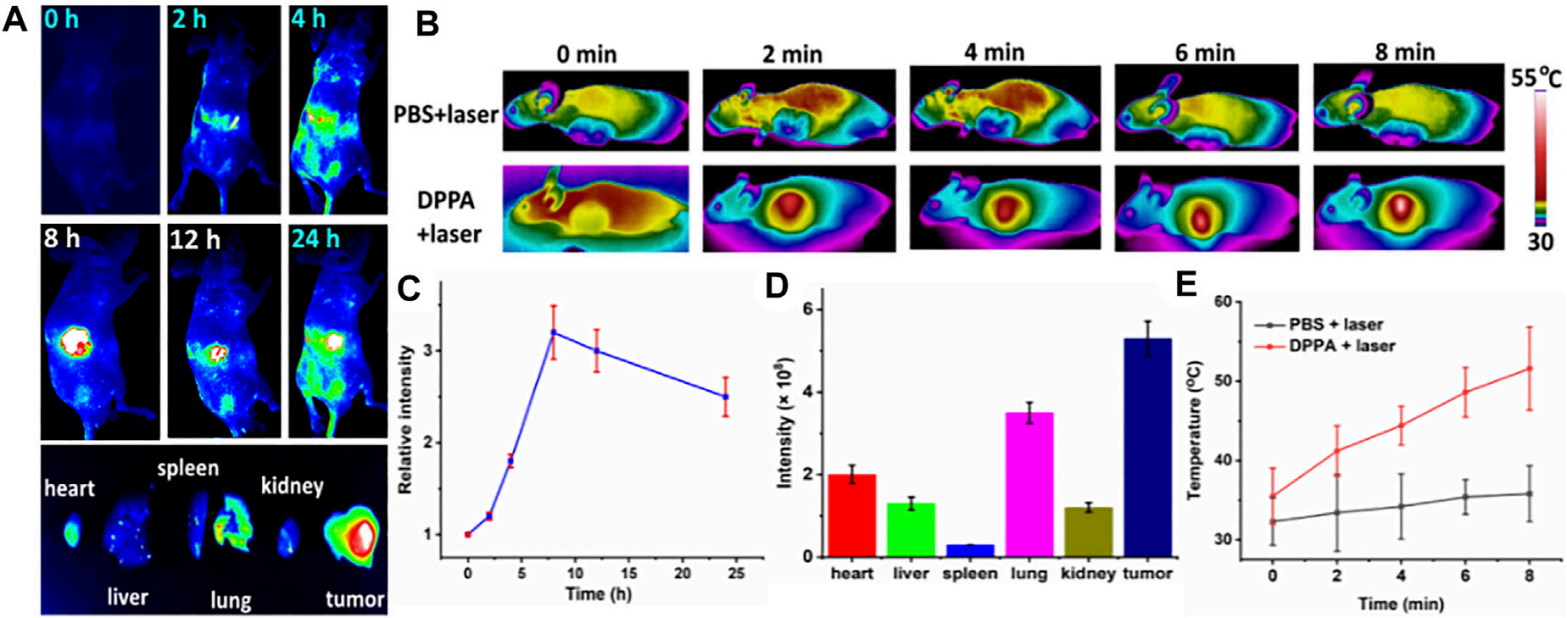
**(A)** Time-dependent fluorescence imaging of A498 tumor with intraveneous injection of DPPA NPs. **(B)** Photothermal imaging of the mouse with an injection of PBS and DPPA NPs (660 nm, 500 W/cm^2^, 8 min). **(C)** Quantification of the tumor intensity at different time points. **(D)** Quantification of the main organs and tumor. **(E)** Tumor temperature change of the PBS + laser and DPPA + laser groups.

Since DPPA NPs exhibit considerable cytotoxicity *in vitro*, we then further investigated the phototherapeutic efficacy *in vivo*. Photothermal and fluorescence imaging were investigated (Figures 4A,B). A total of 15 nude female mice bearing A498 tumor were used in this study. When the tumor volume reached 200 mm^3^, biodistribution was determined by fluorescence imaging *in vivo* after an intravenous injection of DPPA NPs. Time-dependent fluorescence images of the nude mice were captured (Figure 4A). After injection with DPPA NPs for 8 h, the fluorescence intensity of the tumor reached the peak, indicating 8 h is the most appropriate time point for laser performance. After 24 h, these mice were sacrificed. Then, the *ex vivo* fluorescence intensities of the tumor, heart, liver, spleen, lungs, and kidney were recorded (Figure 4C). The fluorescence intensity of the tumor remained the strongest after injection with DPPA NPs for 24 h (Figure 4D). For photothermal imaging, a significant temperature elevation of 16 C was observed for the tumor with laser irradiation for 8 min. In contrast, temperature elevation of the control group is only 4.0 C (Figures 4B,E), which indicates that DPPA NPs show outstanding photothermal efficacy *in vivo*.

**FIGURE 5:**
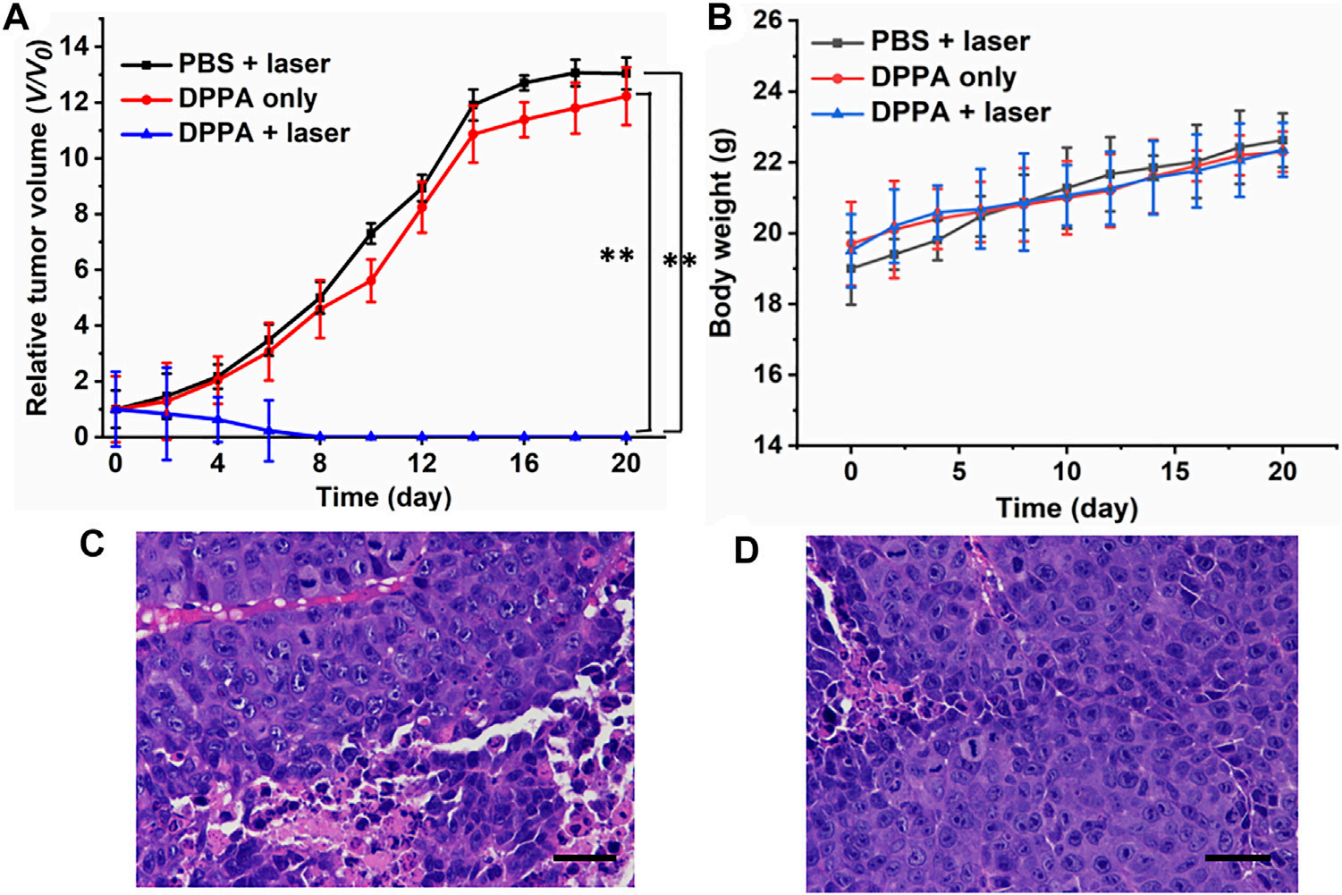
**(A)** Relative tumor volume of the mice in PBS + laser, DPPA-only, and DPPA + laser groups (660 nm, 500 W/cm^2^, 8 min). **(B)** Body weight change. **(D)** H&E stained pictures of the tumor in the **(C)** PBS + laser and **(D)** DPPA-only groups. Scale bar: 10 μm.

The tumor volume of the mice administered with DPPA NPs is parallel to that of the PBS + laser group, suggesting the low dark toxicity of such NPs (Figure 5A). Another piece of evidence is all the mice tend to gain more weight in the three groups, regardless of irradiation or not (Figure 5B). However, tumor proliferation has been suppressed proportionally after laser treatment. After treatment for 3 times, the tumors of the DPPA NPs + laser group completely disappeared, demonstrating the phototherapeutic efficacy of DPPA NPs (Figure 5A). After treatment, the mice were still raised to observe the survival (Figure 5C). Mice in the DPPA NPs + laser group still remained alive while those in the PBS + laser and DPPA NPs-only groups suffered from low survival. Representative mice in the PBS + laser, DPPA NPs-only, and DPPA NPs + laser groups are shown in Supplementary Figure S6. The H&E stained pictures of the tumor in the three groups are very similar with a healthy nucleus (Figure 5D).

**FIGURE 6:**
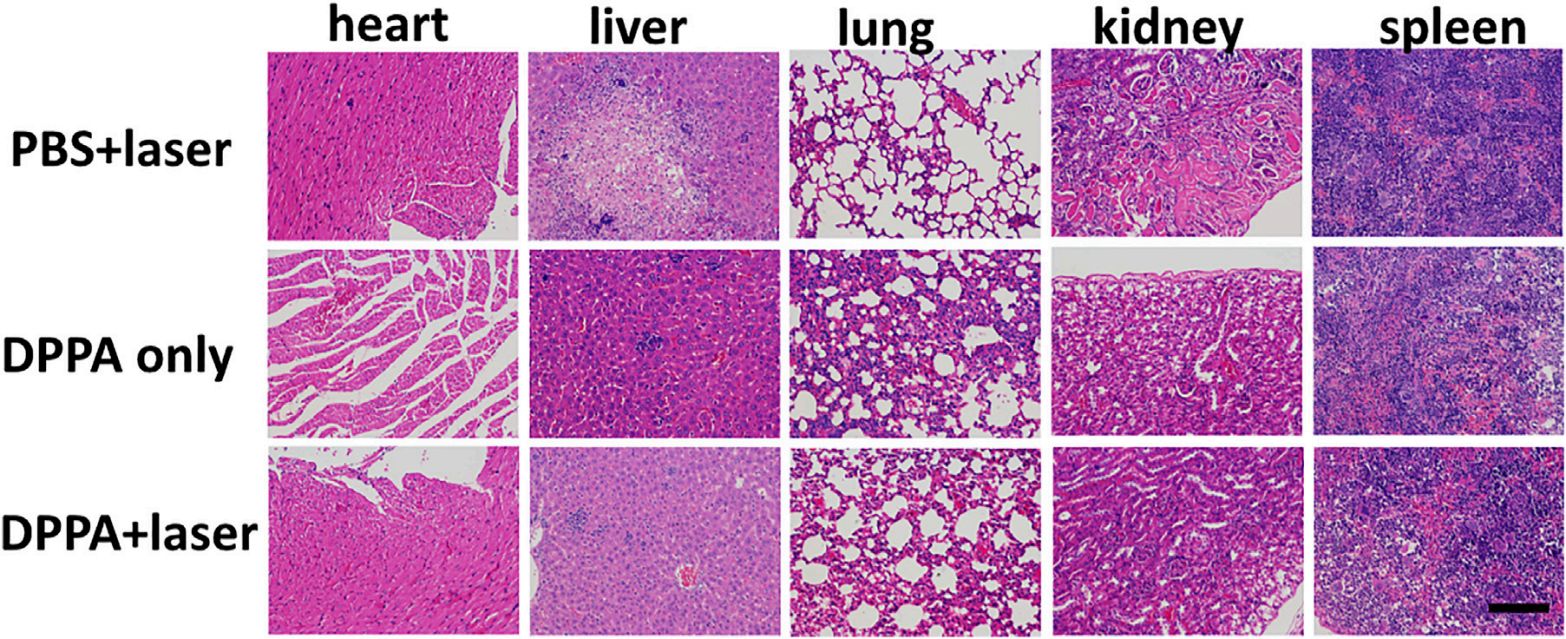
H&E pictures of the heart, liver, lungs, kidney, and spleen in the PBS + laser, DPPA-only, and DPPA + laser groups. Scale bar: 10 μm.

After treatment, all the mice were sacrificed, and the normal organs were collected for the H&E study. No obvious difference was observed in H&E stained pictures (Figure 6). All the results demonstrated that DPPA NPs exhibit strong antitumor activity and low side effects *in vivo*, suggesting their good biocompatibility.

## Conclusion

In summary, an anthracene-functionalized semiconducting photosensitizer DPPA has been designed and prepared with a high ^1^O_2_ QY of 21.3%. The anthracene module acts as a store for fractionated delivery of singlet oxygen because such a module is able to undergo the cycloaddition reaction to store singlet oxygen with laser irradiation while releasing it without irradiation. The as-prepared DPPA NPs still retain the ^1^O_2_ generation ability and simultaneously the high photothermal conversion efficiency (35.6%). MTT assay shows that DPPA NPs show quite low dark toxicity but high phototoxicity with a low IC_50_ of 15.8 μg/ml. *In vivo* photothermal- and fluorescence imaging-guided phototherapy suggest that such NPs are capable of suppressing the tumor growth at a low dose but cause no damage to normal tissues, suggesting the biocompatibility. These results provide some insights to design semiconducting photosensitizers with high phototoxicity, low dark toxicity, and good biocompatibility for photothermal and fractionated photodynamic therapy.

## Data Availability

The original contributions presented in the study are included in the article/Supplementary Material; further inquiries can be directed to the corresponding authors.
