# Supplementary material for: Delivering Singlet Oxygen in Dark Condition With an Anthracene-Functionalized Semiconducting Compound for Enhanced Phototheranostics
Source: Front Bioeng Biotechnol. 2022 Mar 9;10:781766. doi: 10.3389/fbioe.2022.781766 (PMC8959821; doi:10.3389/fbioe.2022.781766)
Supplement: Supplementary file 1 [file DataSheet1.docx]

**Delivering singlet oxygen in dark condition with an anthracene functionalized semiconducting compound for enhanced phototheranostics**

Jian Shen^a^, Lihong Pan^b^, Xujing Zhang^b^, Zhenyuan Zou^b^, Bo Wei^c^, Yongchang Chen*^a^, Xiaoyan Tang*^c^, Dengfeng Zou*^b^

1. *Department of Urology, Changshu No.2 People’s Hospital, Changshu, 215500, Jiangsu, P.R. China. Email:* [*Chenyongchang0327@163.com*](mailto:Chenyongchang0327@163.com)*.*
2. *School of Pharmacy, Guilin Medical University, Guilin, 541004, Guangxi, P.R. China. Email:* [*zdf1226@126.com*](mailto:zdf1226@126.com)
3. *Department of Materials Engineering, Changshu Institute of Technology, Changshu, 215500, Jiangsu, P.R. China. Email:* [*tangxy@cslg.edu.cn*](mailto:tangxy@cslg.edu.cn)

**Preparation of DPPA nanoparticles**

The nanoparticles of DPPA were prepared by nano-precipitation with DSPE-PEG. DSPE-PEG_-2000_ (10 mg) was dissolved in distilled water with ultrasound. And then DPPA (5 mg) was dissolved in tetrahydrofuran (THF, 1 mL). Then 200 μL of such solution was injected into PBS with ultrasound at room temperature. After the mixture was stirred for 10 min, THF was removed by purging nitrogen. The product was then frozen and dried for further use.

**Singlet oxygen generation in DCM and water**

Singlet oxygen quantum yield (^1^O_2_ QY) of DPPA was calculated by using DPBF (1,3-diphenylisobenzofuran) as the ^1^O_2_ indicator. Generally, a mixture was prepared with the absorbance of DPBF being adjusted around 1.0 while that of DPPA being 0.2 to 0.3. After irradiation for different periods of time, the absorbance spectra were recorded. The singlet oxygen quantum yield is calculated according to equation (1)

$$\Phi_{\Delta(DPPA)}=\Phi_{\Delta(MB)}\times\frac{S_{DPPA}}{S_{MB}}\times\frac{F_{MB}}{F_{DPPA}} (1)$$

where *S* stands for the slope of plot of the absorbance of DPBF (at 414 nm) versus irradiation time, and *F* can be calculated by *F*= 1-10^-OD^

$$\Phi_{\Delta(DPPA)}=\Phi_{\Delta(MB)}\times\frac{S_{DPPA}}{S_{MB}}\times\frac{F_{MB}}{F_{DPPA}} (1)$$

The singlet oxygen generation of DPPA NPs in aqueous solution was investigated using SOSG as a probe. A mixture of DPPA NPs (1 μM) and SOSG (10 μM) in water was irradiated and the fluorescence was recorded after different periods of time (0, 1, 2, 3 and 4 min). Singlet oxygen capture was measured by irradiating the mixture and then recording the fluorescence spectra of SOSG without irradiation.

**Photothermal conversion efficiency of DPPA NPs**

DPPA NPs in water was irradiated by laser and then cooled to room temperature. The temperature was recorded by an infrared camera. The photothermal conversion efficiency was calculated according to equation (2-6).

$$\eta=\frac{hs\left( T_{max}-T_{amb} \right)-Q_{Dis}}{I\left( 1-{10}^{-A660} \right)} (2)$$

$$\theta=\frac{T-T_{amb}}{T_{Max}-T_{amb}} \left( 3 \right)$$

$$dt=-\tau s\frac{d\theta}{\theta} \left( 4 \right)$$

$$\tau_{s}=\frac{\sum_{i} m_{i}C_{p,i}}{hs} \left( 5 \right)$$

$$t=-\tau s\ln(\theta) (6)$$

where *h* is the heat transfer coefficient, *S* is the surface area of the container. The *T*_max_ is the highest temperature of DPPA NPs in water at the maximum steady-state temperature, *I* is the laser power density (0.5 W/cm^2^), *A_660_* is the absorbance of the DPPA NPs at 660 nm and *Q_Dis_* is the heat associated with light absorption by the solvent. The variable *τ_s_* is the sample-system time constant, and m_i_ and *C_i_* are the mass and heat capacity of the deionized water (4.2× 10^3^ J/kg^-1^/C^-1^), respectively.

*
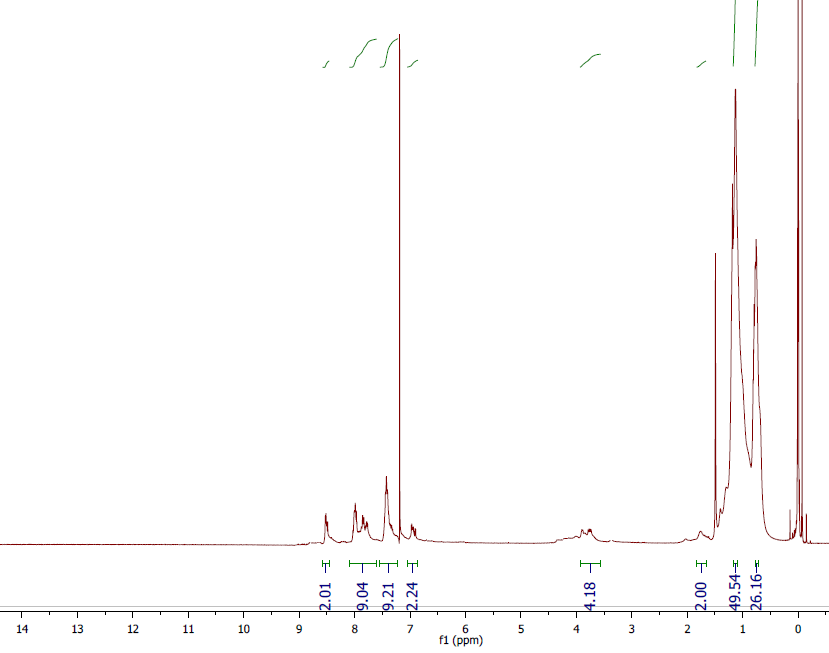
*

Figure S1 ^1^HNMR of DPPA in CDCl_3._


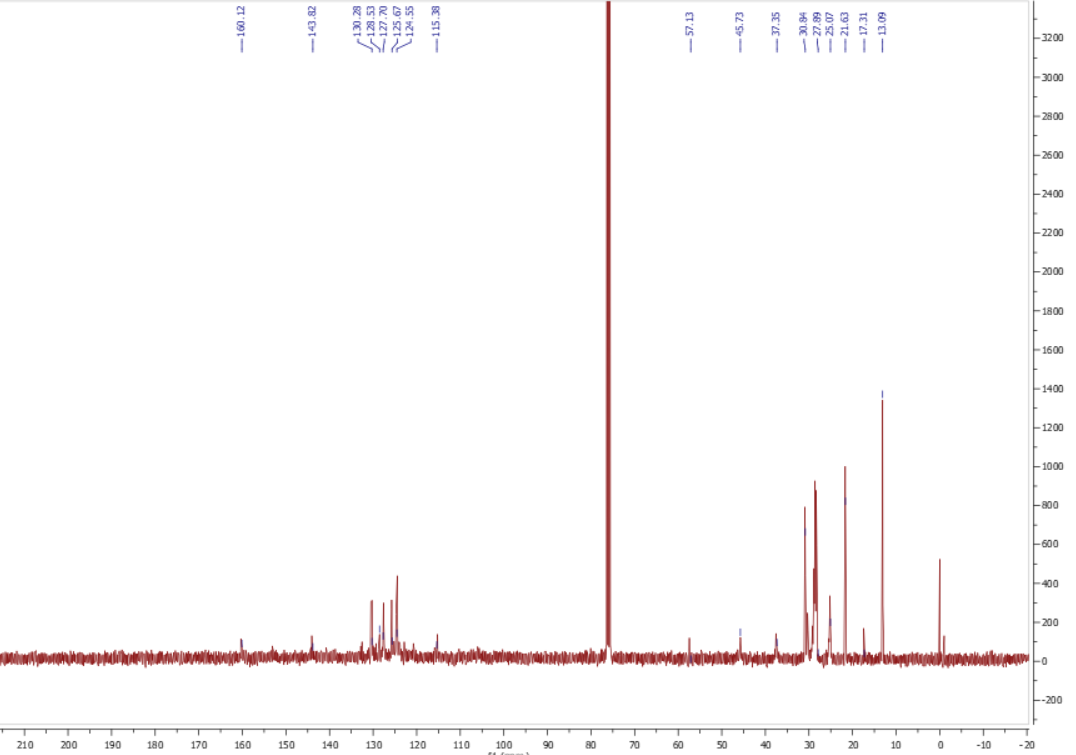


Figure S2 ^13^CNMR of DPPA in CDCl_3._

*
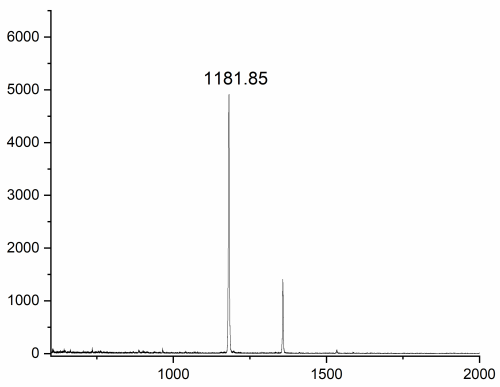
*

Figure S3 Maldi-tof of DPPA


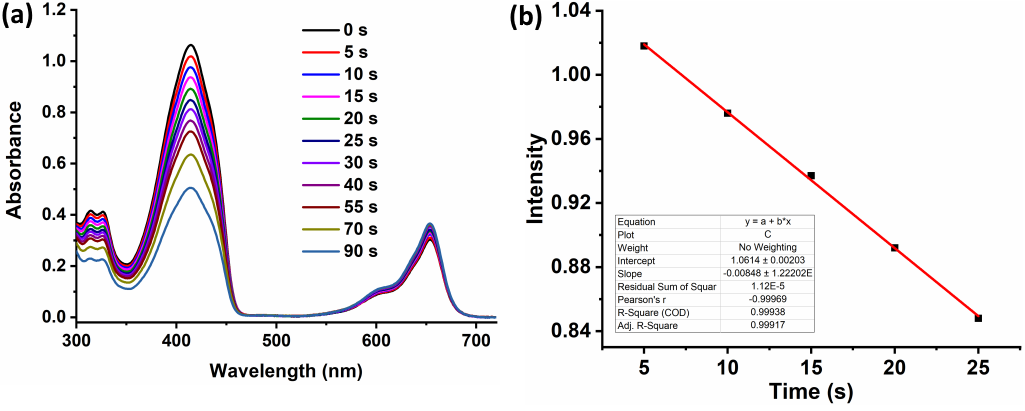


Figure S4 (a) Degradation of DPBF with the presence of MB with irradiation. (b) Linear fitting of the degradation of the absorbance.


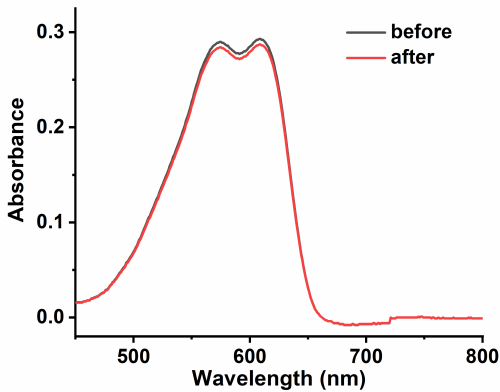


Figure S5 Absorbance of DPPA NPs before and after irradiation.


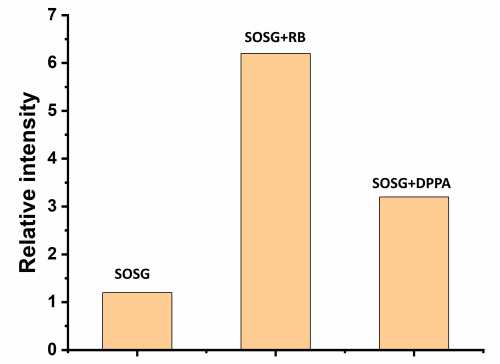


Figure S6 Relative SOSG intensity of the SOSG, SOSG+RB, SOSG+DPPA with laser irradiation.


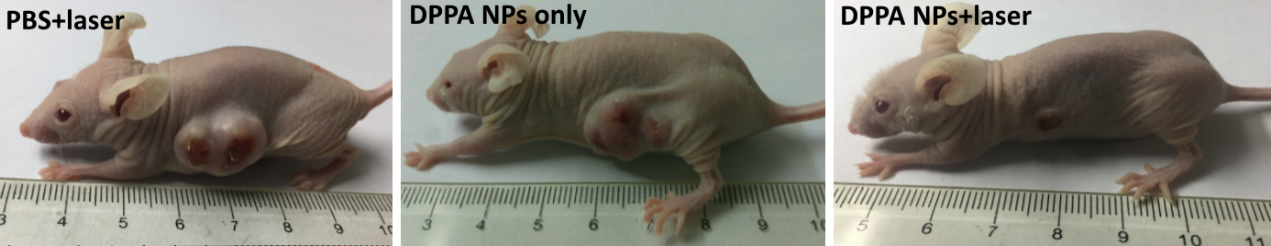


Figure S7 Photograph of representative mice in PBS+laser, DPPA NPs only and DPPA NPs+laser groups.
